# Crystal Structure and Ferromagnetism of the CeFe_9_Si_4_ Intermetallic Compound

**DOI:** 10.1021/acs.inorgchem.3c00547

**Published:** 2023-04-06

**Authors:** Primož Koželj, Stanislav Vrtnik, Justine Boutbien, Jože Luzar, Andreja Jelen, Sorour Semsari Parapari, Pascal Boulet, Marie-Cécile de Weerd, Gwladys Lengaigne, Magdalena Wencka, Vincent Fournée, Julian Ledieu, Sašo Šturm, Janez Dolinšek

**Affiliations:** † J. Stefan Institute, Jamova 39, SI-1000 Ljubljana, Slovenia; ‡ Faculty of Mathematics and Physics, University of Ljubljana, Jadranska 19, SI-1000 Ljubljana, Slovenia; § Institut Jean Lamour, UMR 7198 CNRS − Université de Lorraine, Campus Artem, 2 allée André Guinier, BP 50840, F-54011 Nancy, France; ∥ Institute of Molecular Physics, Polish Academy of Sciences, Smoluchowskiego 17, PL-60-179 Poznan, Poland

## Abstract

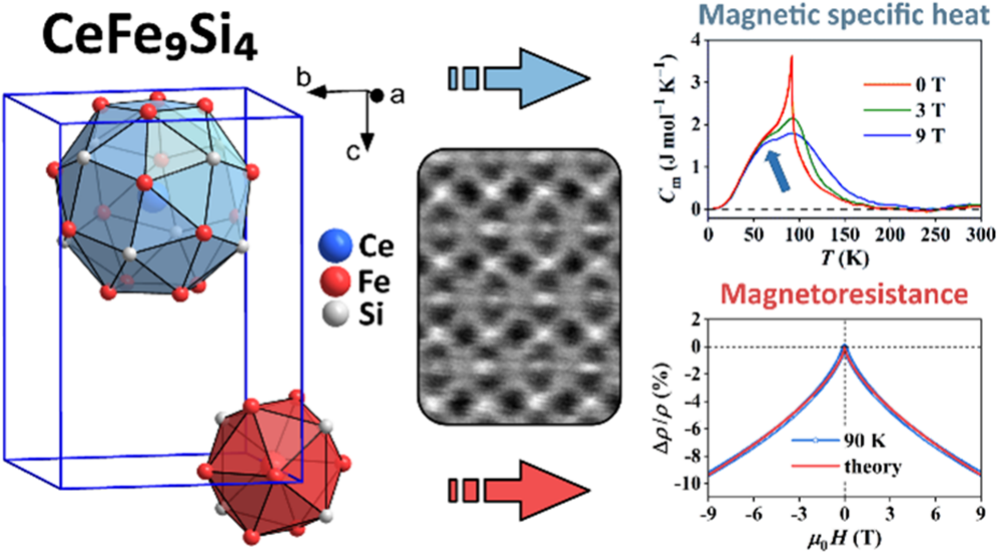

We have determined the crystal structure and the magnetic state of the CeFe_9_Si_4_ intermetallic compound. Our revised structural model (fully ordered tetragonal unit cell, *I*4/*mcm*) agrees with the previous literature report, except for some minor quantitative differences. Magnetically, the CeFe_9_Si_4_ undergoes a ferromagnetic transition at the temperature *T_C_* ≈ 94 K. Ferromagnetism in the combined Ce–Fe spin system is a result of interplay between the localized magnetism of the Ce sublattice and the Fe band (itinerant) magnetism. Ferromagnetic ordering obeys the rather general rule that the exchange spin coupling between atoms possessing more than half-full d shells with atoms possessing less than half-full d shells is antiferromagnetic (where the Ce atoms are considered as light d elements). Since in rare-earth metals from the light half of the lanthanide series, the magnetic moment is directed opposite to the spin, this results in ferromagnetism. The magnetoresistance and the magnetic specific heat show an additional temperature-dependent feature (a shoulder) deep inside the ferromagnetic phase that is considered to originate from the influence of the magnetization on the electronic band structure via the magnetoelastic coupling, which alters the Fe band magnetism below *T_C_*. The ferromagnetic phase of CeFe_9_Si_4_ is magnetically soft.

## Abstract 2

In a search for cerium-containing intermetallic compounds with uncommon magnetic and electrical transport properties, we have synthesized the CeFe_9_Si_4_ compound and determined its crystal structure and magnetic properties. Based on the measurements of magnetization, magnetoresistance, and magnetic specific heat, the compound is a soft ferromagnet below *T_C_* = 94 K.

## 1 Introduction

Many cerium (Ce)-containing intermetallic compounds show uncommon magnetic and electronic transport properties, originating from the fact that the spatial extent of the Ce 4f wave function (containing one electron in the 4f shell) is the largest among the lanthanide series, which makes the exchange interaction between the localized 4f electron and the conduction electrons especially strong. The properties include highly localized magnetism, often with very anisotropic interactions, mixed-valence phenomena, single-ion Kondo effect and Kondo lattice with strongly quenched magnetic moments, heavy-fermion behavior, unconventional superconductivity, and quantum criticality. The Ce compounds and their properties have been reviewed by Sereni.^1^ Interesting Kondo-lattice behavior was reported for the CeNi_9_Si_4_ compound,^2^ which crystallizes in a fully ordered tetragonal (*I*4/*mcm*) variant of the NaZn_13_-type cubic structure. In the CeNi_9_Si_4_, Ni ions do not carry magnetic moments so that the magnetism and the Kondo lattice originate from the Ce sublattice (the La-substituted counterpart LaNi_9_Si_4_ is nonmagnetic^2^). The low-temperature Kondo lattice in the CeNi_9_Si_4_ is considered to be exceptional among the Kondo-lattice systems due to the low Ce fraction of 7 atom % that makes the nearest-neighbor Ce–Ce distances large (5.6 Å) and the Ce–Ce intersite interactions weak. In our research, we have synthesized the CeFe_9_Si_4_ compound, by replacing Ni (that is nonmagnetic in the CeNi_9_Si_4_) with another magnetic transition element Fe. We have characterized the magnetic state of the combined system of localized Ce moments, interacting with the band magnetism of the itinerant Fe moments, by performing measurements of the magnetization, magnetoresistance, and magnetic specific heat. For the CeFe_9_Si_4_ phase, there is already one crystallographic structure report in the literature, obtained in a study of the isothermal section at 900 °C of the Ce–Fe–Si ternary system.^3^ We have redetermined the crystal structure, complemented it with additional information, and compared it with the existing report.^3^

## 2 Material Synthesis and Characterization

The elements Ce of grade 3N, Fe (2N5), and Si (2N5) were alloyed in an arc-melting furnace under 700 mbar Ar atmosphere. The as-cast button was wrapped in a tantalum foil and then annealed in a quartz tube with Ar + 5% H_2_ atmosphere for 10 days@900 °C, followed by 5 days@1140 °C. The final material was polygrain with the grains’ cross dimensions of several 10 μm.

**Figure 1:**
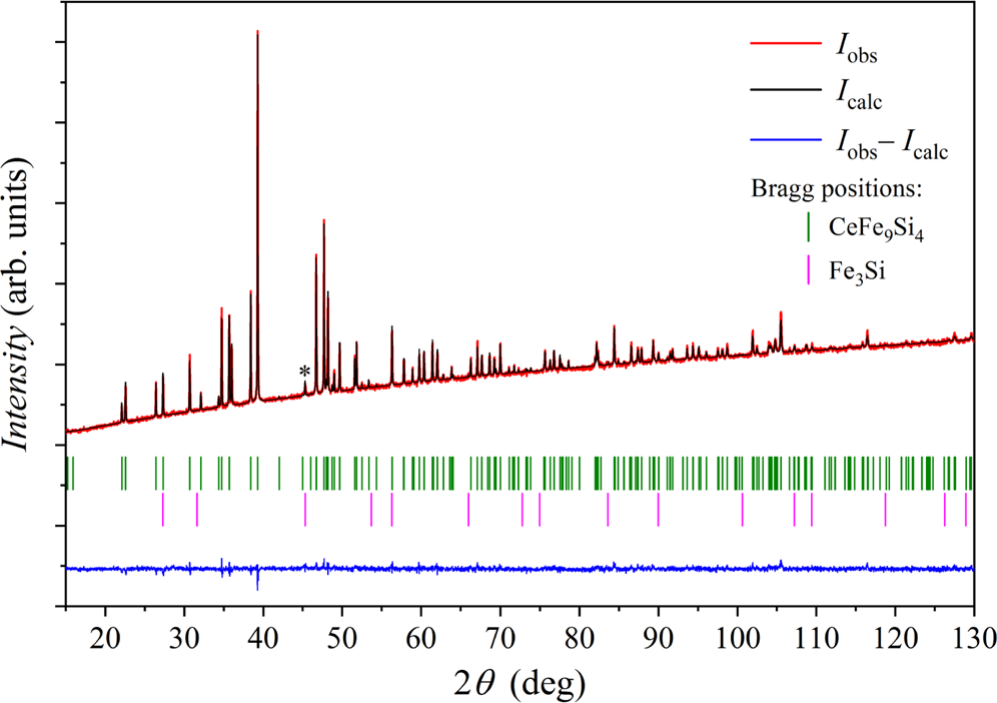
XRD pattern of the CeFe_9_Si_4_ polygrain material with the Rietveld refinement. The upper two traces (overlaid) represent the observed intensity *I*_obs_ (red) and the calculated intensity *I*_calc_ (black), whereas the difference *I*_obs_ – *I*_calc_ is shown in the bottom-most trace (blue). Bragg positions of the CeFe_9_Si_4_ and Fe_3_Si phases are shown as stick spectra. The most intense diffraction peak of the Fe_3_Si impurity phase (at 2θ = 45.24°) is marked by an asterisk.

X-ray diffraction (XRD) powder pattern using Cu Kα_1_ X-ray source is shown in Figure 1 (the XRD experimental details are given in Section 7). The diffraction pattern reveals a tetragonal structure, space group *I*4/*mcm*, and unit cell parameters *a* = 7.8929(1) Å and *c* = 11.6653(2) Å. To check the purity of the investigated CeFe_9_Si_4_ material, we performed Rietveld refinement using Fullprof software.^4^ The result is shown in Figure 1. The Fe_3_Si impurity phase was detected (body-centered cubic, *Fm*3̅*m*, *a* = 5.6644(3) Å)^5,6^ in the amount of 5.7(3) wt %. The most clearly observed diffraction peak of the Fe_3_Si phase is the one at 2θ = 45.24° (marked by an asterisk), while the other peaks are either hidden behind the peaks of the main CeFe_9_Si_4_ phase or are too weak.

**Figure 2:**
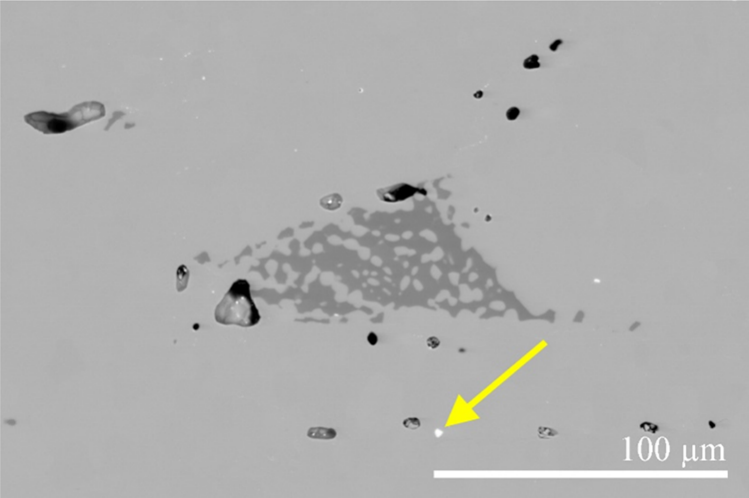
SEM-BSE image of the CeFe_9_Si_4_ material. The matrix is the CeFe_9_Si_4_ phase, the darker-gray precipitates are the Fe_3_Si phase, and the white “spots” (one marked by an arrow) are the CeFe_2_Si_2_ phase. Some porosity is also visible.

A scanning electron microscopy backscattered electron (SEM-BSE) image of the material is shown in Figure 2. The medium-gray matrix contains darker-gray precipitates of several 10-μm cross dimensions and some additional white “spots”. Some porosity, estimated at 0.5% volume fraction, is also visible in the material. The compositions of the three phases were checked by energy-dispersive X-ray spectroscopy (EDS). The composition of the matrix (in atom %, rounded to the nearest integers) is Ce_7_Fe_63_Si_30_, corresponding almost exactly to the stoichiometric formula CeFe_9_Si_4_. The composition of the darker-gray precipitates is Fe_77_Si_23_, corresponding to the phase stoichiometry Fe_3_Si, while the composition of the white spots is Ce_20_Fe_41_Si_39_, corresponding to CeFe_2_Si_2_. The CeFe_2_Si_2_ phase (tetragonal, *I*4/*mmm*)^7,8^ is another impurity in the investigated CeFe_9_Si_4_ material, but its amount is very low, estimated at 0.5 wt %.

**Figure 3:**
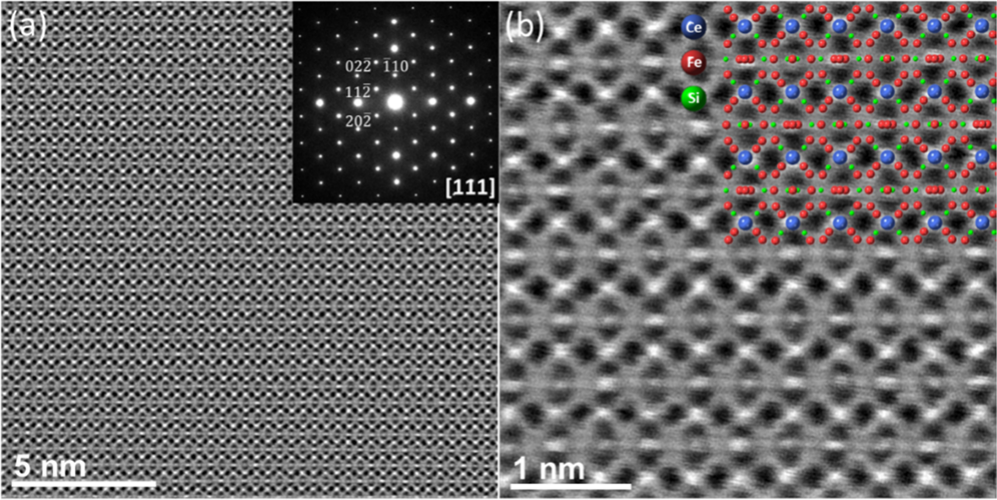
(a) Atomically resolved experimental unprocessed HAADF-STEM image of the CeFe_9_Si_4_ crystal, with a superimposed SAED pattern in the [111] zone axis. (b) High-resolution ABSF-filtered HAADF-STEM image viewed along the [111] crystallographic direction, together with the superimposed atomic model. Blue, red, and green balls represent Ce, Fe, and Si atoms, respectively.

High-angle annular dark-field scanning transmission electron microscopy (HAADF-STEM) experiments were performed to investigate potential planar defects, such as misfits, dislocations, inversion boundaries, etc., at the atomic scale. HAADF-STEM images of the CeFe_9_Si_4_ structure are shown in Figure 3 (experimental details are described in Section 7). In Figure 3a, a representative low-magnification image (often referred to as the *Z*-contrast image), viewed along the [111] crystallographic direction is shown. The analysis of the corresponding experimental selected-area electron diffraction (SAED) pattern in the [111] zone axis (superimposed in Figure 3a) is in agreement with the predicted crystal structure. No planar defects could be detected in the observed specimen region. A representative high-magnification experimental atomic-resolution HAADF-STEM image, viewed along the [111] direction, is shown in Figure 3b. The contrast variation in the atomic-resolution *Z*-contrast images is roughly following a *Z*^2^ dependence on the average atomic number of the probed atom column, which makes the reconstruction of both the atom types and the atomic arrangements of the underlying crystal lattice straightforward. In this regard, the highest-contrast regions in Figure 3b are associated with the fully occupied Ce atomic columns (*Z* = 58), followed by Fe (*Z* = 26) and Si (*Z* = 14) atomic columns, respectively, visualized as lower-intensity regions. The Ce atomic columns are clearly visible in the experimental HAADF-STEM image, while the singular Fe and Si atomic columns cannot be resolved since the small projected distances between the neighboring Fe and Si columns are beyond the given spatial resolution of the electron probe. The constructed CeFe_9_Si_4_ atomic model (as determined by single-crystal XRD, presented in the continuation) viewed along the [111] direction is superimposed on the HAADF-STEM atomically resolved image and fits well with the underlying experimental structure. The reason why the [111] direction, which is quite unusual direction for a tetragonal structure, was chosen is explained in the Supporting Information.

## 3 Structural Model

The structure was determined by single-crystal XRD using a molybdenum Kα X-ray source. The total exposure time was 18.18 h. The frames were integrated with the Bruker SAINT software package using a narrow-frame algorithm. The integration of the data using a tetragonal unit cell yielded a total of 8006 reflections to a maximum θ angle of 24.908°, of which 191 were independent (average redundancy 41.916, completeness 100.0%) and 186 (97.38%) were greater than 2σ(*F*^2^). The crystal structure was solved in the centrosymmetric space group *I*4/*mcm* (No. 140), with the final cell constants *a* = 7.8912(11) Å and *c* = 11.6535(16) Å based upon the refinement of the XYZ-centroids of 7021 reflections above 20 σ(*I*) with 3.497 < θ < 24.908°. Data were corrected for the absorption effects using the multi-scan method (SADABS). The ratio of minimum to maximum apparent transmission was 0.751. The calculated minimum and maximum transmission coefficients (based on the crystal size) were 0.379 and 0.532. All atomic positions were obtained by direct methods (SIR2014) and were in agreement with the positions assigned during the Ce–Fe–Si ternary phase diagram investigations by powder XRD,^3^ showing in particular that all sites are fully occupied without any Fe,Si mixed occupancy. The final reliability factors including the anisotropic thermal factor converged at *R*1 = 0.0062, *w*R**2 = 0.0152 with *I* > 2σ(*I*) and *R*1 = 0.0065, *w*R**2 = 0.0153 with all reflections. The goodness-of-fit (GOF) was 1.13. The largest peak in the final difference electron density synthesis was 0.25 e^–^Å^–3^ and the largest hole was −0.21 e^–^Å^–3^ with an RMS deviation of 0.062 e^–^Å^–3^. On the basis of the final model, the calculated density was 6.912 g cm^–3^ and *F*(000) 1392 e^–^. The above figures did not change when refining the occupancy factors of the atomic sites. This result confirms that the composition of our crystal is in good agreement with the starting composition CeFe_9_Si_4_. The X-ray crystallographic data are summarized in Table 1 (also presented in the Supporting CIF File, deposited in the Cambridge Structural Database as the number CSD 2217058).

**Table 1:** X-ray Crystallographic Data for CeFe_9_Si_4_ (CSD Deposition Number 2217058)

chemical formula	CeFe_9_Si_4_	
formula weight (g mol^–1^)	755.13	
temperature (K)	296(2)	
wavelength (Å)	0.71073 for single-crystal data	
	1.5406 for powder data	

crystal size (μm^3^)	50 × 40 × 30	
crystal system	tetragonal	
space group	*I*4/*mcm* (no. 140)	
unit cell dimensions (Å)	single crystal	powder
	*a* = 7.8912(11)	*a* = 7.8929(1)
	*c* = 11.6535(16)	*c* = 11.6653(2)
Volume (Å^3^)	725.7(2)	726.73(1)
*Z*	4	
calculated density (g cm^–3^)	6.912	
absorption coefficient (mm^–1^)	24.11	
*F*(000)	1392	
θ range for data collection (deg)	3.497–24.908	
index ranges	–8 ≤ *h*, *k* ≤ 8, –12 ≤ *l* ≤ 12	
collected, indep reflns	8006/191	
coverage of the reciprocal sphere (%)	100	
GOF	1.13	
*R* indices (*I* > 2σ(*I*))	*R*(int) = 0.035, *R*1 = 0.0062, *w*R**2 = 0.0152	
all	*R*(int) = 0.035, *R*1 = 0.0065, *w*R**2 = 0.0153	
powder refinement	*R*_B_ = 0.106, *R*_f_ = 0.127	
extinction coeff	0.00042(7)	
number of parameters refined	25	
Δρ_max_, Δρ_min_ (e^–^Å^–3^)	0.25/–0.21	

Solving the structure has generated five independent crystallographic sites in the tetragonal unit cell (Table 2). All sites are fully ordered (occupation 1, no mixed-populated sites), labeled as Ce (Wyckoff position 4a), Fe1 (16l), Fe2 (16k), Fe3 (4d) and Si (16l). The unit cell contains 56 atoms. The isotropic atomic displacement parameters *U*_eq_ of all sites (also given in Table 2) are quite similar. The anisotropic atomic displacement parameters are presented in Table 3.

**Table 2:** Atomic Coordinates and Isotropic Displacement Parameters for CeFe_9_Si_4_ from the Single-Crystal Refinement^a^

atom name	site	*x*	*y*	*z*	*U*_eq_ (Å^2^)
Ce	4a	0	0	1/4	0.00585(11)
Fe1	16l	0.37742(3)	0.12258(3)	0.32124(3)	0.00524(13)
		0.3776(1)	0.1223(2)	0.3218(2)	
Fe2	16k	0.06618(4)	0.20002(4)	0	0.00579(13)
		0.0663(3)	0.2007(3)	0	
Fe3	4d	1/2	0	1/2	0.00486(16)
Si	16l	0.32671(6)	0.17329(6)	0.12219(6)	0.00612(17)
		0.3247(4)	0.1752(4)	0.1212(4)	

a The values obtained from the powder X-ray refinement are given in the second line for the Fe1, Fe2, and Si crystallographic sites.

**Table 3:** Anisotropic Atomic Displacement Parameters (Å^2^) for CeFe_9_Si_4_

	*U*_11_	*U*_22_	*U*_33_	*U*_23_	*U*_13_	*U*_12_
Ce	0.00604(13)	0.00604(13)	0.00549(16)	0	0	0
Fe1	0.00596(16)	0.00596(16)	0.0038(2)	0.00043(9)	–0.00043(9)	–0.00014(14)
Fe2	0.0055(2)	0.0068(2)	0.0050(2)	0	0	–0.00080(14)
Fe3	0.0055(2)	0.0055(2)	0.0035(3)	0	0	–0.0002(3)
Si	0.0067(2)	0.0067(2)	0.0049(3)	–0.00003(18)	0.00003(18)	0.0012(3)

**Figure 4:**
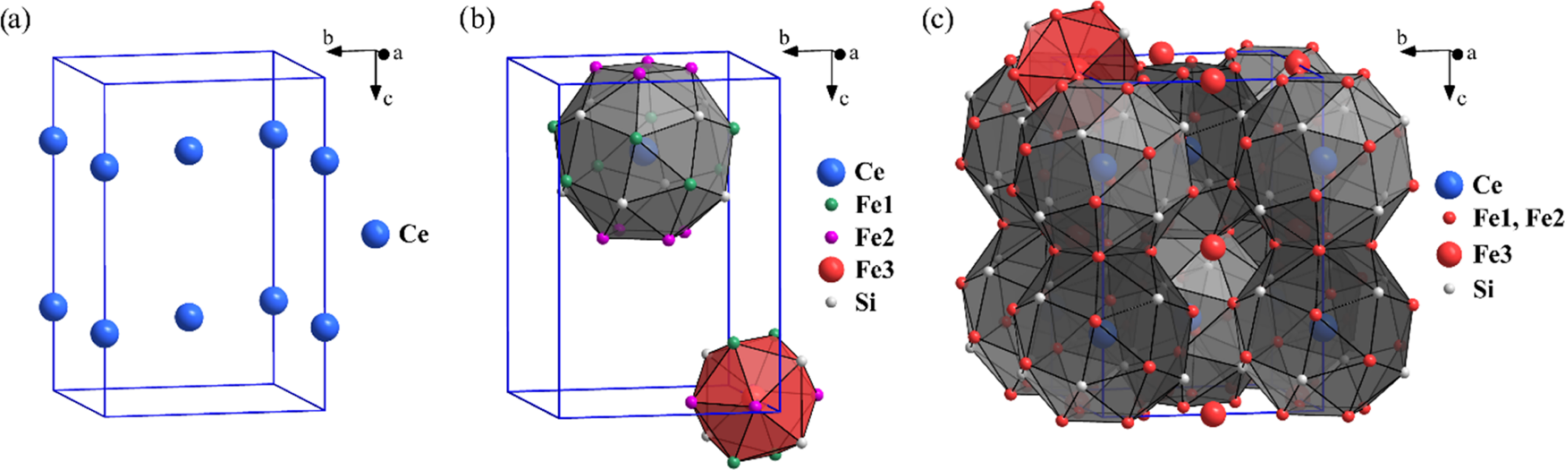
CeFe_9_Si_4_ tetragonal unit cell. (a) Cerium sublattice; (b) one 24-atom polyhedron (the “cage”) around the central Ce atom and one icosahedron around the central Fe3 atom; (c) representation of atomic clusters with regard to the unit cell.

The structure description begins by first considering the Ce sublattice, which is shown in Figure 4a. Adding the Fe and Si atoms, it is apparent that each Ce atom is located in the center of a “cage”, a polyhedron composed of 24 atoms (8 Fe1, 8 Fe2, and 8 Si), distributed on 32 triangles, 4 rectangles, and 2 squares. One such cage is depicted in Figure 4b. The triangles are not equilateral and the two squares (having a tiny rhombic distortion) are formed from the Fe2 atoms. The radial distances between the central Ce atom and the cage atoms are d(Ce–Fe1) = 3.2396 Å, d(Ce–Fe2) = 3.3544 Å, and d(Ce–Si) = 3.2765 Å. The only remaining atoms, which are not incorporated into the cages, are the Fe3 atoms. Each of these is icosahedrally coordinated by four Fe1, four Fe2, and four Si atoms that all already belong to the 24-atom cages. The Fe3 atoms are located in the centers of the icosahedra and the radial distances to the atoms on the icosahedra are d(Fe3–Fe1) = 2.4922 Å, d(Fe3–Fe2) = 2.4242 Å, and d(Fe3–Si) = 2.4016 Å. One such icosahedron is shown in Figure 4b. Note that in Figure 4b, the Fe1, Fe2, and Fe3 atoms are distinguished by different colors and Fe3 is drawn in a larger size. The tetragonal unit cell is depicted in Figure 4c (there, the Fe1, Fe2, and Fe3 atoms are all presented in the same color, but Fe3 has a larger size). The distribution of cages in the unit cell follows the distribution of the Ce atoms. One Fe3-centered icosahedron is also drawn. The type of structure presented in Figure 4 is not unique to the CeFe_9_Si_4_, but was found before in the ACu_9_X_4_ (A = Sr, Ba; X = Si, Ge) series,^9^ LaFe_9_Si_4_,^10^ CeNi_9_Si_4_,^2^ and in the Sn-for-Si substituted rare-earth (RE)-containing compounds RECu_9_Sn_4_ with RE = La to Nd,^11,12^ Eu^12,13^ and Yb.^14^ Comparing the tetragonal unit cell parameters of the CeFe_9_Si_4_ from this work (*a* = 7.8912(11) Å, *c* = 11.6535(16) Å from single-crystal XRD and *a* = 7.8929(1) Å, *c* = 11.6653(2) Å from powder XRD) to the previous report on the same compound^3^ (*a* = 7.892(1) Å, *c* = 11.666(1) Å), the differences are minute. The anisotropic displacement parameters in Table 3 are, however, additionally presented (not included in the crystallographic information in ref (3)).

## 4 Physical Properties

### 4.1 Magnetization versus Temperature

**Figure 5:**
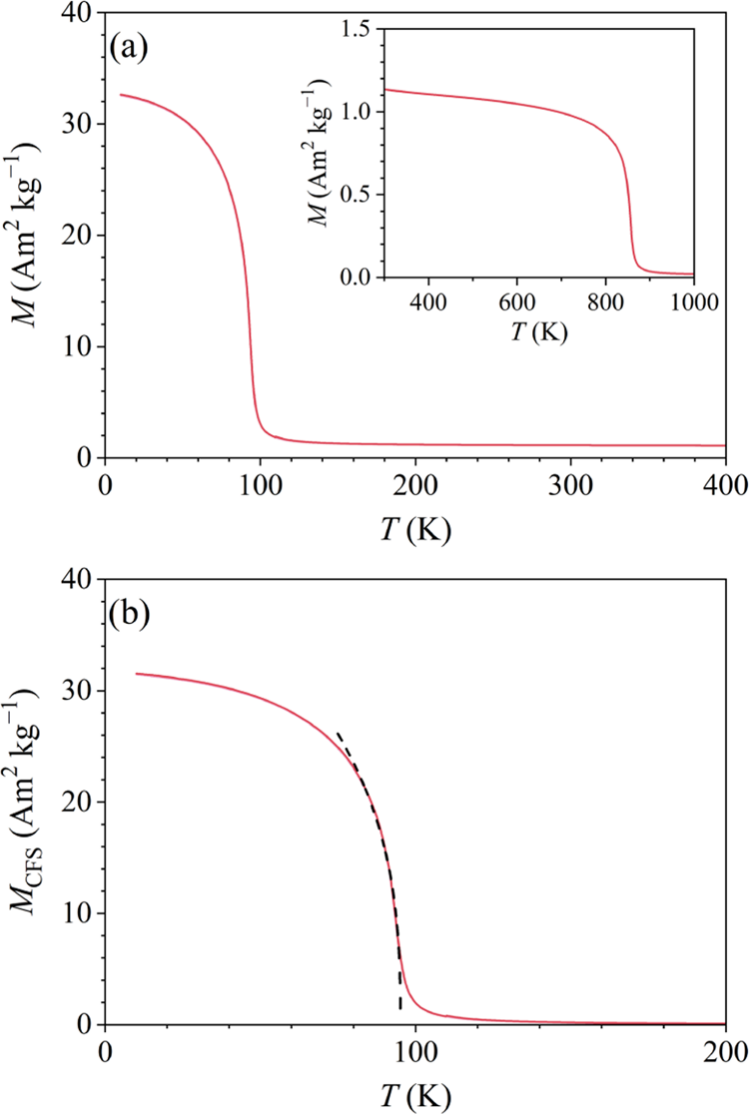
(a) Zfc and fc magnetization curves of the CeFe_9_Si_4_ sample in a magnetic field μ_0_*H* = 0.1 T in the temperature range 10–400 K (*M*_zfc_ and *M*_fc_ are indistinguishable on the graph). The inset shows *M*_zfc_ in the temperature range 300–1000 K. (b) Magnetization of the CeFe_9_Si_4_ phase only (*M*_CFS_), obtained by subtracting the magnetization of the Fe_3_Si impurity phase *M*_FS_ from the total signal below 300 K. Dashed curve is the fit within the critical region with the formula *M*_CFS_ ∝ (*T_C_* – *T*)^β^ (see text).

The direct-current (dc) magnetization of the CeFe_9_Si_4_ sample was first determined in the temperature range 10–400 K using a SQUID magnetometer (experimental details are described in Section 7). The zero-field-cooled (zfc) and field-cooled (fc) magnetization curves in a magnetic field μ_0_*H* = 0.1 T, as a function of temperature, are presented in Figure 5a. A ferromagnetic (FM) transition is observed at *T_C_* ≈ 94 K and the two curves overlap perfectly down to the lowest temperature (i.e., there is no splitting between *M*_zfc_ and *M*_fc_). The small positive, constant-like offset of the baseline above *T_C_* (of magnitude about 1.2 Am^2^ kg^–1^, in mass magnetization units) that persists up to 400 K indicates that this signal could originate from another FM fraction in the sample.

To find out the origin of this “high-temperature” FM signal, we have repeated the *M*(*T*) measurement in the same field, this time using a furnace operating between 300 and 1000 K. The *M*(*T*) curve is shown in the inset of Figure 5a, where an FM transition at about 840 K is evident. This transition temperature is the same as the FM transition temperature reported in the literature for the Fe_3_Si compound.^5^ Since the Fe_3_Si is present as an impurity phase in the investigated CeFe_9_Si_4_ material (in the amount of 5.7 wt %), it is straightforward to assign the high-temperature FM signal to the Fe_3_Si inclusions (the second impurity phase, CeFe_2_Si_2_ at 0.5 wt %, is a Pauli paramagnet^7,8^ and contributes negligibly to the total signal). At 300 K, the magnitude of the Fe_3_Si signal amounts to *M*_FS_ = 1.13 Am^2^ kg^–1^, accounting well for the baseline offset above *T_C_* in the main panel of Figure 5a. The total magnetization of the sample (CeFe_9_Si_4_ phase plus Fe_3_Si inclusions) at 10 K in a 0.1 T field amounts to *M*_CFS_ + *M*_FS_ = 33 Am^2^ kg^–1^ and since the *M*_FS_ signal is practically constant below room temperature, it constitutes about 3% of the total magnetization at 10 K. For the analysis of the CeFe_9_Si_4_ FM phase, we have subtracted the constant offset (*M*_FS_) from the total signal in the low-temperature range below 300 K, and the resulting CeFe_9_Si_4_-only magnetization *M*_CFS_ is shown in Figure 5b. The magnetization within the critical temperature region just below *T_C_* was fitted with the formula *M*_CFS_ ∝ (*T_C_* – *T*)^β^ (dashed curve in Figure 5b), yielding the critical exponent β = 0.36.

### 4.2 Magnetization versus Magnetic Field

**Figure 6:**
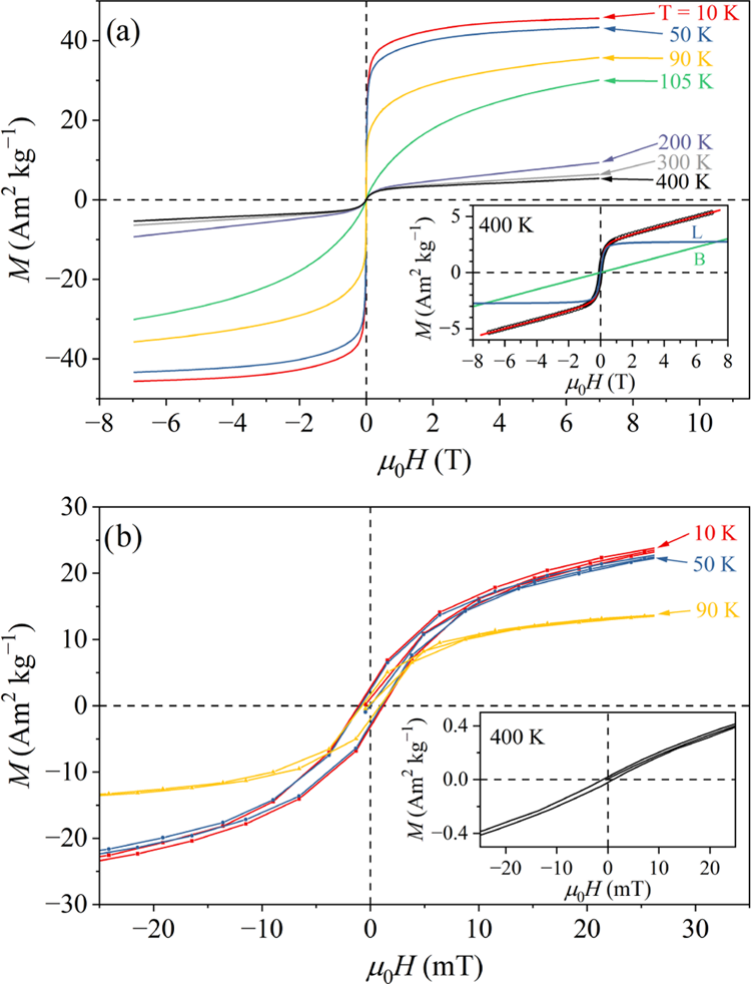
(a) *M*(*H*) curves of the CeFe_9_Si_4_ sample at temperatures between 10 and 400 K. The inset shows the *M*(*H*) curve at 400 K on an expanded vertical scale, together with the fit *M* = *M*_CFS_ + *M*_FS_ (red curve) described in the text (*M*_CFS_ is the magnetization of the CeFe_9_Si_4_ phase, and *M*_FS_ is the magnetization of the Fe_3_Si impurity phase). The *M*_CFS_ paramagnetic contribution to the total fit marked with B (standing for “Brillouin”) and the *M*_FS_ FM contribution marked with *L* (standing for “Langevin”) are also shown. (b) *M*(*H*) curves at temperatures 10, 50, 90, and 400 K on an expanded scale about the origin, to show the hysteresis.

The magnetization versus the magnetic field, *M*(*H*), curves of the CeFe_9_Si_4_ sample were measured in the field range ±7 T. The *M*(*H*) curves at temperatures between 10 and 400 K are shown in Figure 6a. Two types of the *M*(*H*) curves are evident, corresponding to the temperature regions above and below *T_C_* ≈ 94 K. Below 94 K, the curves are typical ferromagnetic, with a very rapid increase in the low-field region around *H* = 0 and practically no hysteresis (to be discussed later), indicating a magnetically soft material. The saturation magnetization at *T* = 10 K in a 7-T field amounts to *M*^sat^ = 46 Am^2^ kg^–1^. We keep in mind that this is the total saturation magnetization of the CeFe_9_Si_4_ phase plus the Fe_3_Si inclusions. Above 94 K, the CeFe_9_Si_4_ matrix is in the paramagnetic phase, while the Fe_3_Si inclusions are in the FM phase. This is mirrored in the shape of the *M*(*H*) curves, which are at temperatures considerably higher than 94 K (i.e., at 200, 300, and 400 K in Figure 6a) a sum of a term linear in *H*, corresponding to the paramagnetic magnetization of the CeFe_9_Si_4_ phase and an FM term due to the Fe_3_Si inclusions. The field dependence of the CeFe_9_Si_4_ paramagnetic magnetization is in fact given by the Brillouin function, which reduces to a linear-in-*H* dependence at high temperatures.

The *T* = 400 K *M*(*H*) curve is shown on an expanded scale in the inset of Figure 6a, together with the fit . Here, *k*μ_0_ is the paramagnetic susceptibility of the CeFe_9_Si_4_ phase, *M*_FS_^sat^ is the saturation magnetization of the Fe_3_Si inclusions, and  is the Langevin function, with *x* = μ_0_μ*H*/*k*_B_*T*. Within the Langevin model (which is suitable to reproduce the FM-type *M*(*H*) curves, but not their hysteresis), the magnetic moment μ is a classical vector that can assume any value (μ → ∞), accounting for the large group magnetic moment of the FM phase. The fit-determined parameters are *k* = 0.38 Am^2^ kg^–1^ T^–1^, *M*_FS_^sat^ = 2.8 Am^2^ kg^–1^, and μ = 7319μ_B_, where μ_*B*_ is the Bohr magneton. Knowing *M*_FS_^sat^ at 400 K (and assuming that this value does not change much down to 10 K), we are able to estimate the saturation magnetization of the CeFe_9_Si_4_ phase as *M*_CFS_^sat^ = *M*^sat^ – *M*_FS_^sat^ ≈ 43 Am^2^ kg^–1^. Recalculating this value in units of μ_B_ per formula unit (f.u.), i.e., per one CeFe_9_Si_4_ “molecule”, we obtain *M*_CFS_^sat^ = 5.7 μ_B_/f.u., accounting for the total magnetic moment of one Ce ion and nine Fe. The above value can be contrasted to the estimated maximum possible magnetic moment of one formula unit (μ_f.u._), by taking literature values of the saturation moment of a Ce^3+^ ion μ_Ce_ = 2.14μ_B_^15^ and ionic values of the iron moments (μ_Fe^3+^_ = 5.9 μ_B_, μ_Fe^2+^_ = 5.4 μ_B_),^16^ yielding μ_f.u._ in the range 50–55 μ_B_. Using the ionic values of the iron moments implicitly assumes that they are localized (like in an insulator) so that the much smaller experimental value of 5.7 μ_B_ is a strong indication of itinerant (band) magnetism of the Fe subsystem in the CeFe_9_Si_4_. The moments of the Ce sublattice are, however, localized.

Searching for the magnetization hysteresis, palladium correction was applied to the *M*(*H*) curves to remove the small hysteresis of the superconducting magnet of the employed SQUID magnetometer. The *M*(*H*) curves at temperatures 10, 50, 90, and 400 K are presented in Figure 6b on an expanded scale about the origin. A tiny hysteresis with the same coercive field of μ_0_*H*_c_ = 1.0 ± 0.1 mT is observed at all temperatures, also at 400 K that is high above the FM transition in the CeFe_9_Si_4_. This indicates that the hysteresis originates from the Fe_3_Si impurity phase and confirms the practically ideal magnetic softness of the CeFe_9_Si_4_ FM phase.

### 4.3 Electrical Resistivity and Magnetoresistance

**Figure 7:**
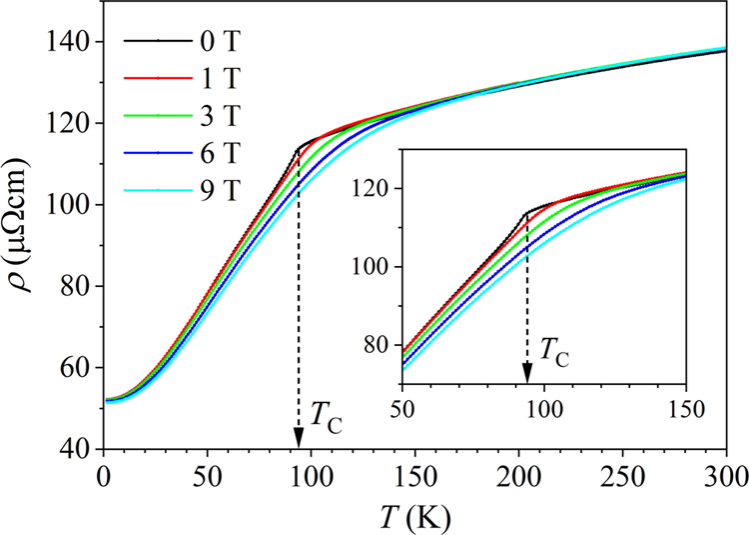
Electrical resistivity of the CeFe_9_Si_4_ sample at temperatures between 2 and 300 K in selected magnetic fields in the range μ_0_*H* = 0–9 T (values indicated on the graph). Expanded portions of the resistivity curves around *T_C_* are shown in the inset.

The electrical resistivity of the CeFe_9_Si_4_ sample at temperatures between 2 and 300 K in the magnetic field range μ_0_*H* = 0–9 T is shown in Figure 7. The resistivity ρ(*H*,*T*) increases with temperature and is field-independent in the limits much below and much above the FM transition temperature *T_C_* ≈ 94 K, while it is strongly field-dependent in the region of *T_C_*. The 2-K residual resistivity amounts to ρ_2K_ = 52 μΩcm, while the room-temperature resistivity is ρ_300K_ = 137 μΩcm. In zero magnetic field, the resistivity shows a discontinuous change of slope at *T_C_*, where the slope is larger below *T_C_* and smaller above. In an increasing magnetic field, the discontinuous change of slope at *T_C_* becomes rounded and smeared and the resistivity continuously decreases within the *T_C_* region. Such temperature and field dependence of the resistivity is typical of a conducting ferromagnet. According to the Matthiessen’s rule, the resistivity is a sum of a temperature-independent term ρ_0_ due to the elastic scattering of conduction electrons by quenched impurities (this term determines the low-temperature residual resistivity), the temperature-dependent term due to phonon scattering ρ_ph_ that increases with temperature as *T*^*n*^ (where *n* = 1 well above the Debye temperature and *n* = 3–5 far below it) and the FM-specific term ρ_FM_ due to spin-disorder scattering so that the total resistivity is ρ = ρ_0_ + ρ_ph_ + ρ_FM_. The origin of ρ_FM_ is the magnetic scattering of conduction electrons by the fluctuating spins that participate in the FM ordering. Upon approaching the *T_C_*, the fluctuating spins create a spin-dependent potential of random character that fluctuates between values differing by the on-site exchange interaction and scatters the conduction electrons. Within the FM phase, the spin-disorder resistivity is given by^17−19^
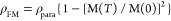
1where *M* is the spontaneous magnetization of the FM state and ρ_para_ is the resistivity of the paramagnetic state above *T_C_*. ρ_para_ is temperature-independent and proportional to the square of the exchange coupling constant between the localized spins and the conduction electrons. According to eq 1, ρ_FM_ is constant above *T_C_* and starts to drop below, giving a discontinuous change of slope in the total ρ at *T_C_*. Applying a magnetic field within the *T_C_* region where the magnetic susceptibility is high, *M* is increased, thus producing a negative magnetoresistance. All this is consistently observed in the experimental ρ(*H*,*T*) curves of Figure 7. Spin-disorder scattering is pronounced in RE metals and alloys. In the CeFe_9_Si_4_, it is reasonable to consider that it originates mainly from the scattering of conduction electrons by the fluctuating Ce^3+^ moments.

**Figure 8:**
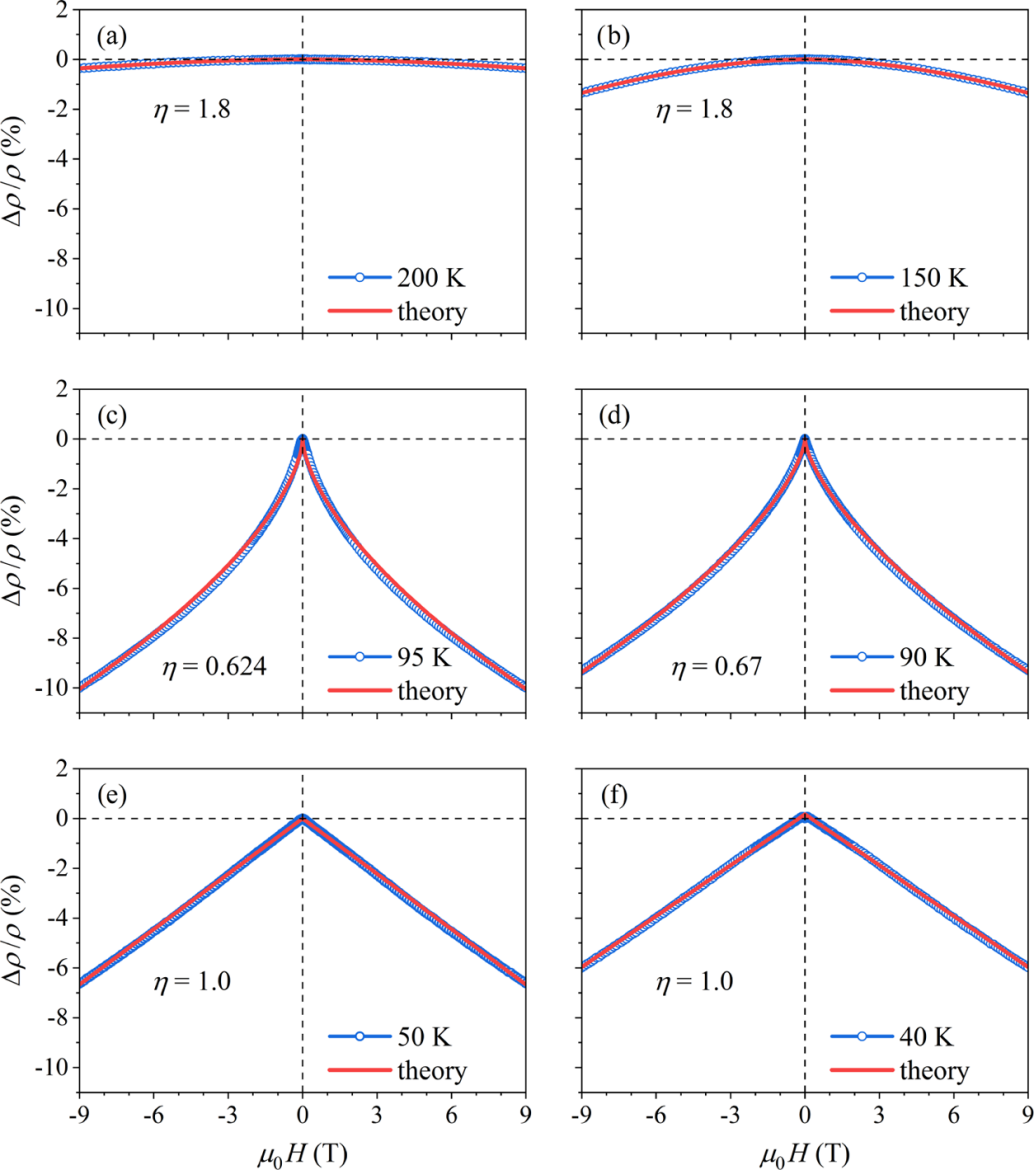
Magnetoresistance curves, Δρ/ρ, of the CeFe_9_Si_4_ sample in the magnetic field range μ_0_*H* = ± 9 T in three characteristic temperature regimes relative to *T_C_* ≈ 94 K. The curves at *T* = 200 K (a) and 150 K (b) belong to the high-temperature regime *T* > *T_C_*; the curves at *T* = 95 K (c) and 90 K (d) are taken in the close vicinity of the FM phase transition (the *T* ≈ *T_C_* regime); the curves at *T* = 50 K (e) and 40 K (f) are taken deep inside the FM phase (*T* < *T_C_*). Solid curves are fits with the mean-field theory, described in the text. The values of the power-law fit parameter η are indicated in the graphs.

The magnetoresistance [ρ(*H*) – ρ(0)]/ρ(0) = Δρ/ρ is another suitable quantity to characterize the magnetic state of a metallic alloy. The magnetoresistance curves of the CeFe_9_Si_4_ sample in the magnetic field range μ_0_*H* = ±9 T in three characteristic temperature regimes relative to *T_C_* ≈ 94 K are shown in Figure 8. The Δρ/ρ curves at *T* = 200 and 150 K (Figure 8a,b) belong to the high-temperature regime *T* > *T_C_*, corresponding to the part of the paramagnetic phase, where short-range ordered spin clusters start to form on approaching the *T_C_*. The curves at *T* = 95 and 90 K (Figure 8c,d) are taken in the close vicinity of the FM phase transition (the *T* ≈ *T_C_* regime), whereas the curves at *T* = 50 and 40 K (Figure 8e,f) are taken deep inside the FM phase (*T* < *T_C_*). All Δρ/ρ curves are negative, which is a characteristic feature of the FM-type magnetoresistance, while their magnitude and shape vary according to the temperature regime.

Theoretical expressions for the FM magnetoresistance within the above three characteristic temperature regimes are known in the literature. The calculations were first performed in the mean-field theory^20^ and then improved by a more involved formalism.^21^ Our experimental Δρ/ρ curves presented in Figure 8 could be well reproduced theoretically already by the simpler mean-field theory, which we discuss in the following. The mean-field predictions are Δρ/ρ ∝ −*H*^2^/(*T* – *T_C_*)^2^ at *T* > *T_C_*, Δρ/ρ ∝ −*H*^2/3^ at *T* ≈ *T_C_*, and  at *T* < *T_C_* (the more involved theory^21^ brings for the *T* ≈ *T_C_* and *T* < *T_C_* regimes an extra logarithmic term that multiplies the mean-field result, while the mean-field result for *T* > *T_C_* remains unchanged). The fits of the experimental magnetoresistance curves in Figure 8 were all performed with the expression Δρ/ρ = −const.*H*^η^, by treating the power-law exponent η as a free parameter at each measured temperature. The fits of the paramagnetic phase magnetoresistance yielded the exponent η = 1.8 for both measured temperatures of 200 and 150 K (solid curves in Figure 8a,b), which is fairly close to the mean-field value of 2. The small difference could be due to the fact that the mean-field result is valid in the weak-field limit, whereas the experiments were done up to 9 T, which can no more be considered as a weak field. The magnetoresistance very close to *T_C_* was reproduced well with the fit parameter η = 0.62 at 95 K and η = 0.67 at 90 K (solid curves in Figure 8c,d), where the second value (0.67) is exactly the mean-field result of 2/3. The magnetoresistance below *T_C_* was reproduced well with the exponent η = 1.0 (equal to the mean-field result) at both temperatures 50 and 40 K (solid lines in Figure 8e,f). The analysis at still lower temperatures was not done, because the mean-field theory is known to become inadequate in the *T* → 0 limit.^20^

**Figure 9:**
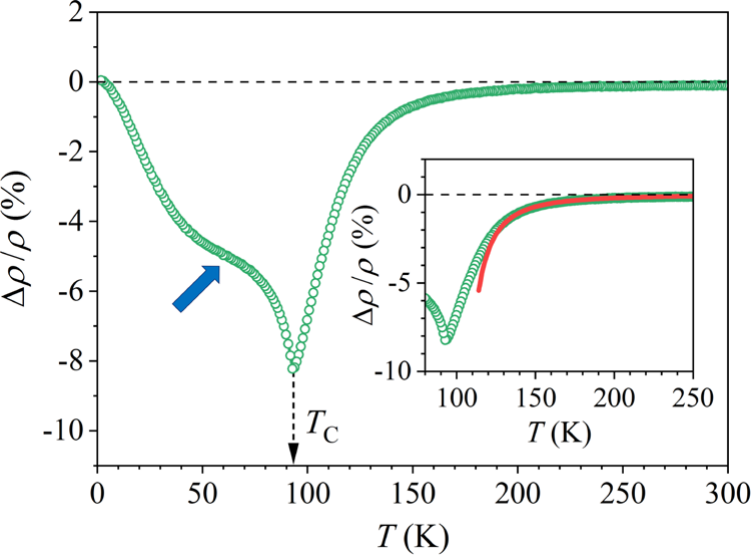
Magnetoresistance of the CeFe_9_Si_4_ sample in a constant magnetic field μ_0_*H* = 6 T, measured as a function of temperature in the interval 2–300 K. The shoulder inside the FM phase is marked by an arrow. The inset shows the parabolic fit Δρ/ρ = −2170K^2^/(*T* – *T*_*C*_)^2^ with *T*_C_ = 94 K (red solid curve) that reproduces well the paramagnetic phase data in the temperature range 130–250 K.

The magnetoresistance as a function of temperature in the interval 2–300 K, measured in a constant magnetic field μ_0_*H* = 6 T is shown in Figure 9. Upon cooling from 300 K, the magnetoresistance in the paramagnetic phase becomes nonzero at about 210 K and then decreases (its absolute value |Δρ/ρ| increases) upon approaching the *T_C_*, reaching the minimum in Δρ/ρ (or the maximum in |Δρ/ρ|) at *T_C_* ≈ 94 K. Below *T_C_*, |Δρ/ρ| starts to decrease upon cooling, until it becomes zero in the *T* → 0 limit. The decrease of |Δρ/ρ| within the FM phase upon cooling is not a simple monotonous one, but a broad shoulder is observed at about 60 K (marked by an arrow in Figure 9).

The mean-field predictions for the temperature dependence of the magnetoresistance are Δρ/ρ ∝ −1/(*T* – *T_C_*)^2^ in the paramagnetic phase (*T* > *T_C_*), a minimum in Δρ/ρ at *T_C_*, and  in the FM phase (*T* < *T_C_*). Far away from *T*_*C*_, the magnetoresistance approaches zero on both sides of the temperature axis (at *T* ≫ *T_C_* and *T* ≪ *T_C_*) and the minimum value is reached at *T_C_*, which is all consistently observed in the experimental magnetoresistance of Figure 9. A parabolic magnetoresistance Δρ/ρ ∝ −1/(*T* – *T_C_*)^2^ is also observed experimentally in the paramagnetic phase high above *T_C_*, as verified in the inset of Figure 9, where the parabolic fit reproduces well the experimental data in the temperature range 130–250 K. The experimental magnetoresistance at *T* < *T_C_* (in the FM phase), however, does not follow the mean-field result , which predicts a simple monotonous decrease of |Δρ/ρ| upon cooling below *T_C_*, because the experimental curve exhibits additional shoulder deep inside the FM phase. Theoretically, such shoulder cannot be derived even by the more involved theory of the FM magnetoresistance^21^ so that it is a genuine property of the combined Ce–Fe ferromagnetic spin system in the CeFe_9_Si_4_ crystal. We shall show that the same anomaly (the shoulder) is observed also in the magnetic specific heat.

### 4.4 Magnetic Specific Heat

The specific heat *C* of a conducting ferromagnet is a sum of the electronic (*C*_el_), lattice (*C*_latt_), and magnetic (*C*_m_) terms, *C* = *C*_el_ + *C*_latt_ + *C*_m_. The electronic term *C*_el_ = γ*T*, where γ is the electronic specific heat coefficient, shows linear-in-*T* dependence up to high temperatures (such as the melting point of the alloy). The lattice contribution is written at low temperatures (typically below 10 K) within the Debye model as *C*_latt_ = α*T*^3^, where the lattice specific heat coefficient α is related to the Debye temperature θ_D_ via the relation θ_D_ = (12π^4^*R*/5α)^1/3^ and *R* is the gas constant. Since the Debye temperature is usually slightly temperature-dependent, this is the low-temperature value of θ_D_. The FM contribution at low temperatures can also be written in a simple form, *C*_m_ = δ*T*^3/2^, representing the contribution due to spin-wave excitations (magnons) of the FM spin system. The total low-temperature specific heat of a ferromagnet is given by^22^
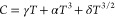
2which is dominated by the electronic term because of its lowest power *T* dependence. The magnetic specific heat *C*_m_ vanishes in the *T* → 0 limit. While the magnon specific heat represents the FM contribution to the total specific heat at low temperatures, there is a much larger magnetic specific heat *C*_m_ in the region of the FM phase transition (around *T_C_*), where spin ordering results in a decrease of the magnetic energy that is released from the spin system as heat and detected as an exothermic peak (an anomaly) at *T_C_* in the total specific heat. We have extracted this magnetic specific heat by a procedure described in the following.

**Figure 10:**
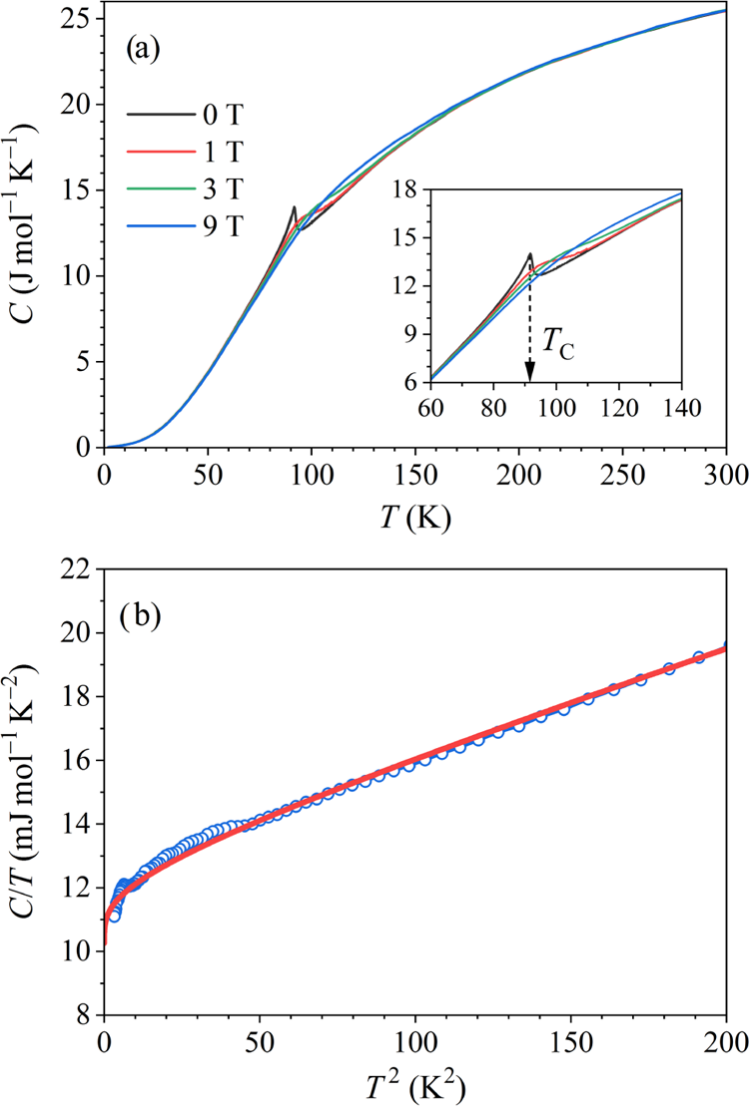
(a) Total specific heat *C* of the CeFe_9_Si_4_ sample in the temperature range 2–300 K in selected magnetic fields between 0 and 9 T (values indicated on the graph). The inset shows the curves on an expanded scale within the phase-transition region. (b) Low-temperature zero-field specific heat in a *C*/*T* vs *T*^2^ plot. The red solid curve is the fit with eq 2, and the fit parameters are given in the text.

The total specific heat of the CeFe_9_Si_4_ sample in the temperature range 2–300 K, measured in magnetic fields between 0 and 9 T is shown in Figure 10a in a *C* vs *T* plot. In zero field, a sharp anomaly (a peak) is observed at about 92 K, corresponding to the FM transition temperature *T_C_* (this *T_C_* value is about 2 K lower than the one determined from the magnetization and magnetoresistance). In an increasing field, the anomaly broadens and smears toward higher temperatures, which is better evident in the inset of Figure 10a, where the curves are presented on an expanded scale within the phase-transition region. The low-temperature zero-field specific heat in a *C*/*T* vs *T*^2^ plot is shown in Figure 10b, together with the fit with eq 2. The down-curvature of the experimental data (and the fit) in the *T* → 0 limit is due to the magnon contribution. The fit parameters are γ = 10.2 mJ mol^–1^ K^–2^, δ = 0.95 mJ mol^–1^K^–5/2^, and θ_D_ = 406 K (recall that this is the low-temperature θ_*D*_ value). The above γ value is close to the one of the Ce metal (γ_Ce_ = 12.8 mJ mol^–1^ K^–2^), while the γ value of iron (γ_Fe_ = 4.9 mJ mol^–1^ K^–2^) is considerably smaller (Si is a semiconductor).^22^

**Figure 11:**
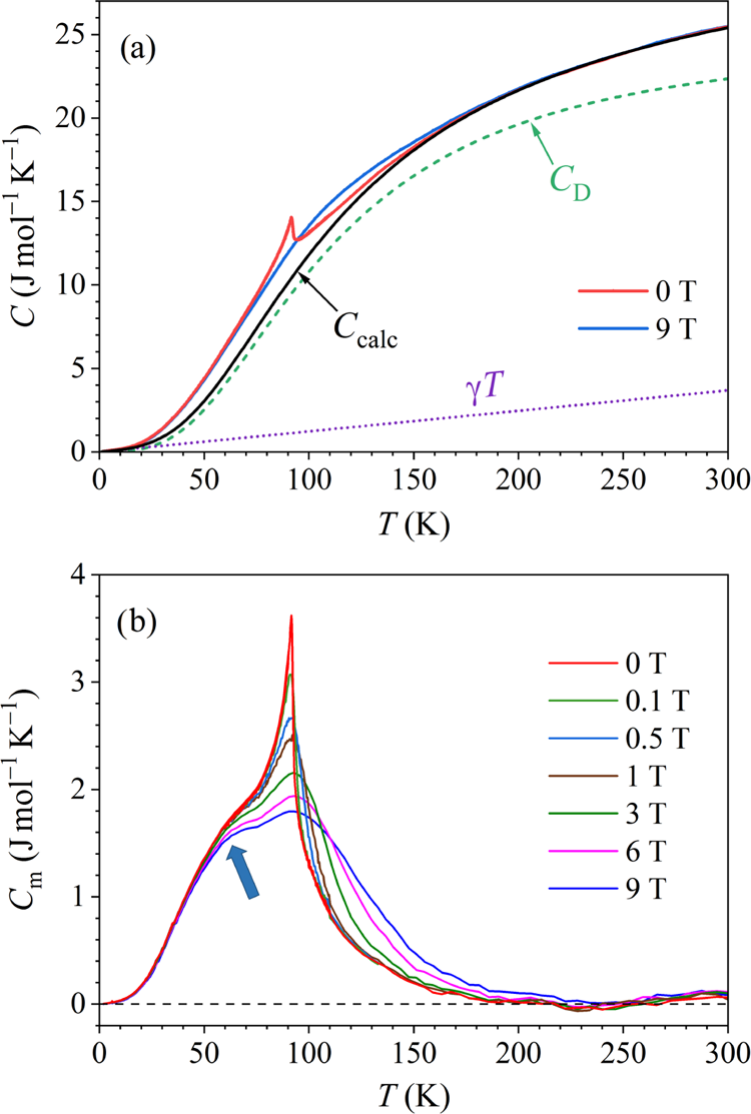
(a) Experimental total specific heat *C* in zero- and 9-T magnetic fields, together with the calculated specific heat *C*_calc_ = γ*T* + *C*_D_ in the temperature range 2–300 K. The electronic (γ*T*) and Debye (*C*_D_) contributions are also shown separately (details are described in the text). (b) Magnetic specific heat *C*_m_ = *C* – γ*T* – *C*_D_ in different external magnetic fields (values indicated on the graph). The shoulder at about 60 K is indicated by an arrow.

The magnetic specific heat *C*_m_ was extracted from the total specific heat *C* by treating the lattice contribution theoretically within the Debye model in the entire investigated temperature range using the formula^22^
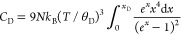
3Here, *N* is the number of atoms in the crystal, *x* = *ℏ*ω/*k*_B_*T*, ω is the frequency of the lattice vibrations, and *x*_D_ = θ_D_/*T*. It is known that for many solids the Debye model reproduces well the experimental data at low temperatures *T* < θ_D_/50 (i.e., below about 2% of the Debye temperature, typically below 10 K) and at high temperatures *T* > θ_*D*_/2 (typically above 150–200 K). We have further assumed that *C*_m_ = 0 high in the paramagnetic phase (above 200 K, as estimated from the magnetoresistance) and very small in the *T* → 0 limit. In the next step, we have calculated the specific heat *C*_calc_ = γ*T* + *C*_D_ in the entire investigated temperature range 2–300 K, by adjusting the θ_D_ fit parameter while keeping the γ value determined before from the fit in Figure 10b, to match *C*_calc_ to the experimental zero-field specific heat *C* in the high-temperature regime between 180 and 300 K. The theoretical *C*_calc_ obtained with the fit parameter value θ_D_ = 450 K (the high-temperature θ_D_ value), together with the separated electronic (γ*T*) and Debye (*C*_D_) contributions is presented in Figure 11a. The experimental total specific heat *C* in zero magnetic field and in a 9-T field is also shown, where excellent matching to *C*_calc_ in the interval 180–300 K is evident. The agreement of *C*_calc_ with *C* in the low-temperature limit *T* → 0 is also good (*C*_calc_ and *C* are both very small there so that any mismatch between them is not visible on the graph).

The magnetic specific heat was obtained by subtraction, *C*_m_ = *C* – γ*T* – *C*_D_, and the procedure was repeated for the experimental specific heat measured in all magnetic fields. The graphs of *C*_m_(*H*,*T*) in the temperature range 2–300 K for all (seven) measured magnetic fields are shown in Figure 11b. The zero-field magnetic specific heat *C*_m_(0,*T*) shows a sharp peak at *T_C_* ≈ 92 K, which broadens and shifts to higher temperatures in an increasing magnetic field. This is the known smearing of the FM phase transition by the external magnetic field. The *C*_m_(*H*,*T*) curves show, however, an additional feature at lower temperatures deep inside the FM phase. The zero-field *C*_*m*_(0,*T*) exhibits a shoulder at about 60 K (marked by an arrow), which develops into a peak in an increasing field that largely overlaps with the main peak at 92 K (best seen on the 9-T curve). The origin of the shoulder will be discussed in the Section 5. Here, we note that this shoulder is in agreement with the shoulder observed in the temperature-dependent 6-T magnetoresistance presented in Figure 9, occurring at practically the same temperature. We also emphasize that the Debye specific heat *C*_*D*_ and the electronic specific heat γ*T* are both smooth functions of the temperature in the entire investigated temperature range so that the shoulder in *C*_*m*_ could not be artificially created by the subtraction procedure, but is indeed a genuine property of the combined Ce–Fe spin system.

## 5 Discussion

Since the CeFe_9_Si_4_ contains the RE element Ce from the light half of the lanthanide series and the magnetic element Fe from the heavy half of the 3d series, ferromagnetism of this compound is not surprising. It can be predicted from the more general rule that exchange spin coupling between atoms possessing more than half-full d shells with atoms possessing less than half-full *d* shells is antiferromagnetic.^23^ Due to the electronic configuration 4f*^n^*5d^1^6s^2^, the RE atoms are in this context considered as light d elements. The coupling of spins of the ferromagnetic 3d transition elements TM = Fe, Co, and Ni to the spins of the RE elements is therefore antiparallel. However, in light RE metals, the magnetic moment is mainly orbital in character and directed opposite to the spin according to Hund’s third rule. Consequently, though the spins of the RE and TM atoms are antiparallel, their magnetic moments are parallel, resulting in ferromagnetism. Examples of the RE–TM ferromagnetic compounds that conform to this rule are the widely commercially used permanent magnets SmCo_5_ and Nd_2_Fe_14_B (and their derivatives), in which the RE moments are localized, while the 3*d* moments are itinerant.^24^

The FM transition temperature *T*_*C*_ ≈ 94 K of the CeFe_9_Si_4_ is considerably higher than the *T*_*C*_s of isotypic ferromagnetic RE-containing compounds without a magnetic 3d transition element. Examples are the RECu_9_Sn_4_ (RE = Ce, Pr, Nd, and Eu) compounds with *T*_*C*_s of 5.5, 10.5, 15, and 10.5 K, respectively.^11−13^ The magnetism of these compounds originates solely from the RE atoms and the relatively low *T*_*C*_s are considered to be related to the large nearest-neighbor RE–RE distances of about 6 Å, which make the indirect exchange (Ruderman–Kittel–Kasuya–Yosida, RKKY) interaction between the localized RE moments weak. In the CeFe_9_Si_4_, there are two sources of magnetism, the localized Ce^3+^ 4f moments and the itinerant Fe 3d moments. The Ce–Ce nearest-neighbor distances of about 5.6 Å are also large so that the RKKY interaction between the Ce moments cannot account for the strong increase of *T*_*C*_. Instead, the higher *T*_*C*_ of CeFe_9_Si_4_ is the effect of iron. The type of exchange interaction between the localized Ce and the delocalized Fe moments (either direct exchange or RKKY indirect exchange) is a subtle question because it depends on the degree of the Fe moments delocalization. However, since the Ce distances to the Fe1 and Fe2 atoms that constitute the 24-atom cage around the Ce atom are considerably shorter (d(Ce–Fe1) = 3.24 Å, d(Ce–Fe2) = 3.35 Å), the Ce–Fe exchange interactions must be stronger, shifting the *T*_*C*_ of CeFe_9_Si_4_ to higher temperatures. A quantitative theoretical treatment of magnetism of the combined Ce–Fe system with localized Ce moments and delocalized Fe moments in the presence of three kinds of exchange interactions (Ce–Ce, Ce–Fe, and Fe–Fe) is very complex and remains a challenge for future studies.

The fact that the small, temperature-independent hysteresis in the *M*(*H*) curves of the CeFe_9_Si_4_ sample was experimentally observed also at 400 K (high above the FM transition temperature *T*_*C*_ ≈ 94 K) strongly suggests that the observed hysteresis originates from the Fe_3_Si impurity phase, while the CeFe_9_Si_4_ phase possesses vanishing small magnetic anisotropy and is hence magnetically soft. The reason for the vanishing anisotropy is not obvious. The magnetocrystalline anisotropy is straightforward to discuss for the RE ions, which possess well-localized 4*f* magnetic electrons. The electric charge eρ_4f_(*r⃗*) of the Ce^3+^ 4f shell is not spherically symmetric, but oblate (flattened). It is coupled to the electrostatic potential φ_cf_ (*r⃗*) of the surrounding crystal charges, resulting in the crystal-field anisotropy energy ε_a_ = ∫eρ_4f_(*r⃗*)φ_cf_(*r⃗*)d^3^*r*. Expanding the 4f charge density in spherical harmonics, the leading term in ε_*a*_ is the coupling of the ρ_4*f*_ quadrupole moment *Q*_2_ (which is negative for the Ce^3+^ oblate ion) to the electric field gradient (EFG) tensor created by the crystal charge distribution at the cerium site. For the tetragonal symmetry of the unit cell, the EFG tensor *V*_*ij*_ in the crystal-fixed reference frame (*a*,*b*,*c*) is diagonal, having the eigenvalues *V*_*aa*_ = *V*_*bb*_ = −*V*/2 and *V*_*cc*_ = *V*. This makes the *c* crystallographic direction (the tetragonal axis) the easy direction of magnetization, with anisotropy to the (*a*,*b*) tetragonal plane. At low temperatures, higher moments (hexadecapole and 64-pole moments, *Q*_4_ and *Q*_6_) of the 4f charge density become also important and the anisotropy energy for the tetragonal symmetry depends both on the polar angle θ between the magnetization and the tetragonal (easy) direction and the azimuthal angle ϕ in the tetragonal plane. There is, therefore, no *a priori* reason for the vanishing magnetocrystalline anisotropy based on symmetry considerations of the tetragonal unit cell. Strong magnetocrystalline anisotropy of the RE magnetization, with the easy axis along [001] and hysteretic *M*(*H*) curve was indeed observed experimentally in the RECu_9_Sn_4_ (RE = Ce, Pr) compounds,^12^ suggesting that the Ce fraction of magnetization in the CeFe_9_Si_4_ should also be anisotropic and introduce some hysteresis in the total magnetization (Ce + Fe) of this phase, in contrast to the experimental finding that the phase is magnetically soft. The Fe itinerant magnetism in the CeFe_9_Si_4_ phase is obviously soft, while there can be various reasons why the anisotropic magnetism of the Ce subsystem has not been observed experimentally in the total magnetization. One possibility is a significant reduction of the Ce magnetic moment by the crystal fields, which would happen when the ground state doublet of the three crystal-field-split doublets of the Ce^3+^*J* = 5/2 states would be populated only, the other two being too high in energy. The second possibility follows from the analogy to the strongly anisotropic magnetism in the related CeCu_9_Sn_4_ single crystal.^12^ There, the Ce magnetization within the FM phase for the field along the [001] easy axis saturates at 0.1 T and the saturated moment reaches 1.75 μ_*B*_ (slightly reduced with respect to the Ce^3+^ free-ion saturated value of 2.14 μ_B_, very likely due to the crystal fields), while the magnetization for the field along the [100] perpendicular direction is almost negligibly small (verified up to 1 T). For a polygrain material with randomly oriented microcrystallites (like our investigated CeFe_9_Si_4_ sample), such anisotropic magnetization can average to a quite small value and becomes experimentally unobservable in the presence of another (larger) source of magnetization, like the magnetically soft Fe magnetization.

We discuss next the possible origin of the shoulder that appears in the magnetic specific heat and in the temperature-dependent magnetoresistance curve inside the FM phase much below the main peak at *T*_*C*_. In the case of the magnetic specific heat, the shoulder reflects an increasing FM spin order in the combined Ce–Fe spin system upon cooling below *T*_*C*_, which is manifested as an increased heat release from the system due to the lowering of the magnetic energy. It is well known that the FM-ordered moments in a crystal may distort the lattice and change the lattice parameters via the magnetoelastic coupling. This is the phenomenon of volume magnetostriction,^25^ a temperature-dependent effect that is proportional to the square of the spontaneous magnetization, *M*(*T*)^2^. The changed lattice parameters reflect the modified distances between the atoms in the unit cell, which in turn change the electronic band structure, the exchange coupling, the magnetic dipole interactions, and the crystal fields. Ferromagnetic ordering may thus have a profound effect on the electronic, magnetic, thermal, and optical properties of a solid. In the CeFe_9_Si_4_ compound, it is plausible to consider that the growing magnetization of the Ce–Fe system below *T*_*C*_ imposes a temperature-dependent change of the electronic band structure that alters the band magnetism of the itinerant Fe moments in a way to further increase the FM ordering. This produces a shoulder in the magnetic specific heat deep inside the FM phase. The increased FM order similarly affects the magnetotransport, because larger magnetization increases the (absolute) magnetoresistance. Since the temperature evolution of the shoulder in the magnetoresistance curve of Figure 9 is very much the same as that of the shoulder in the magnetic specific heat in Figure 11b, the two shoulders obviously share common origin, considered to be the magnetization-induced change of the electronic band structure that alters the Fe band magnetism. The above considerations offer a plausible hypothesis, which still needs to be proven by the electronic band structure calculations for the CeFe_9_Si_4_ structure in the presence of magnetoelastic effects. Such demanding ab initio calculations go beyond the scope of this paper.

Finally, we comment on the influence of the Fe_3_Si and CeFe_2_Si_2_ impurity phases in the investigated CeFe_9_Si_4_ material on the analysis and properties of the CeFe_9_Si_4_ ferromagnetic phase. The content of the CeFe_2_Si_2_ Pauli-paramagnetic impurity phase (about 0.5 wt %) is so low that it cannot significantly influence the magnetism of the CeFe_9_Si_4_ phase and can hence be neglected. The Fe_3_Si phase is present in a more significant amount (5.7 wt %) and its ferromagnetism with *T*_*C*_ ≈ 840 K was indeed experimentally observed in the temperature-dependent magnetization and the *M*(*H*) curves of the investigated CeFe_9_Si_4_ material, but due to the very high value of the *T*_*C*_, the magnetization *M*_*FS*_ is practically constant in the temperature range of the FM phase in the CeFe_9_Si_4_ (below *T*_*C*_ ≈ 94 K) and could be reliably subtracted from the total *M*(*T*) magnetization, to yield the magnetization *M*_CFS_ of the CeFe_9_Si_4_ phase only. Proper identification of the *M*_*FS*_ could also be done in the *M*(*H*) curves. For the magnetoresistance, the presence of the two impurity phases is unimportant, because the precipitates are well isolated in space from each other and do not yield a connected path in the material from one to the other electrode to allow current flow via the impurity phases. The specific heat within the experimentally studied temperature range below 300 K is also practically insensitive to the presence of the impurity phases, due to their low amount and the high Curie temperature of the Fe_3_Si phase. The performed analysis of ferromagnetism in the CeFe_9_Si_4_ phase should hence be correct.

## 6 Conclusions

We have determined the crystal structure of the CeFe_9_Si_4_ intermetallic compound and found that the revised structural model agrees with the previous one,^3^ with some minute quantitative differences. Cerium atoms are located in the centers of highly symmetric cages, the 24-atom polyhedra composed of the Fe1, Fe2, and Si atoms, while the Fe3 atoms are located in the centers of icosahedra composed of the Fe1, Fe2, and Si atoms that already belong to the 24-atom cages. This type of structure is not unique to the CeFe_9_Si_4_ compound but was found before in the ACu_9_X_4_ (A = Sr, Ba; X = Si, Ge) series, LaFe_9_Si_4_, CeNi_9_Si_4_ and RECu_9_Sn_4_ (RE = La, Ce, Pr, Nd, Eu, and Yb).

The measurements of magnetization, magnetoresistance, and magnetic specific heat have revealed that CeFe_9_Si_4_ undergoes a ferromagnetic transition at the Curie temperature *T*_C_ ≈ 94 K. Ferromagnetism in the combined Ce–Fe spin system is a result of an interplay between the localized magnetism on the Ce sublattice and the Fe band (itinerant) magnetism. It follows from the general rule that the exchange spin coupling between atoms possessing more than half-full d shells with atoms possessing less than half-full d shells is antiferromagnetic (where the Ce atoms are considered as light d elements). Since in light RE metals, the magnetic moment is directed opposite to the spin, this results in ferromagnetism. The magnetoresistance and the magnetic specific heat show an additional temperature-dependent feature (a shoulder) deep inside the ferromagnetic phase that is considered to originate from the influence of the magnetization on the electronic band structure via the magnetoelastic coupling, which alters the Fe band magnetism below *T*_C_ in a temperature-dependent manner. This hypothesis still needs to be proven by the electronic band structure calculations in the presence of magnetoelastic effects. The FM phase of the CeFe_9_Si_4_ is magnetically soft. Unlike the isostructural CeNi_9_Si_4_ compound, which shows Kondo lattice behavior and Ni is in a nonmagnetic state, Fe is magnetic in the CeFe_9_Si_4_ compound, leading to ferromagnetism of the combined Ce–Fe spin system.

## 7 Experimental Section

Powder XRD was performed using a Bruker D8 Advance diffractometer in Bragg–Brentano θ/2θ configuration. The wavelength was a Johansson Ge(111) monochromated Cu K*α*_1_ (λ = 1.54056 Å). The sample was finely ground in an agate mortar and deposited in zero-background sample holder. The data were collected with a 0.021° step 2θ width and 4 s counting time per point over the 2θ range from 10 to 130°. The sample was allowed to rotate perpendicular to the diffusion plane at a speed of 15 rev/min. The detection was performed using a LynxEye PSD detector in one-dimensional (1D). The analysis of the diffraction patterns was performed by Rietveld profile refinement using the FullProf and WinPlotr software packages.^4,26,27^

Single-crystal XRD data were collected on a Bruker Kappa Apex II diffractometer equipped with a mirror monochromator and a Mo Kα microsource (λ = 0.71073 Å). The Apex2 program package was used for the cell refinement and data reduction. The structure was solved by using direct methods and refined with the SHELXL-2014 program package.^28^ Semiempirical absorption correction (SADABS) was applied to the data.

SEM-BSE imaging and EDS chemical composition determination were performed by a scanning electron microscope ThermoFisher Quanta 650 ESEM equipped with EDS Oxford Instruments, AZtecLive, Ultim Max SDD 40 mm^2^.

HAADF-STEM imaging was performed by a JEM-ARM 200F Cold FEG TEM/STEM transmission electron microscope operating at 200 kV, coupled with a GIF Quantum 965 ER and equipped with a spherical aberration (Cs) probe and image correctors (point resolution 0.12 nm in the TEM mode and 0.078 nm in the STEM mode). Atomically resolved STEM images were processed using an Average Background Subtraction Filter (ABSF)^29^ to increase the signal-to-background ratio. The collection semiangle for the HAADF-STEM detector was set between 68 and 280 mrad. For the TEM specimen preparation, a CeFe_9_Si_4_ sample was cut into blocks and mounted face-to-face in a brass ring of 3 mm in diameter with epoxy glue. The mounted sample was then mechanically ground to a thickness of 100 μm and dimpled down to 15 μm at the disc center (Dimple grinder, Gatan Inc., Warrendale, PA). The TEM specimen was finally ion-milled (PIPS, Precision Ion Polishing System, Gatan Inc., Warrendale, PA) using 3 kV Ar^+^ ions at an incidence angle of 8° until perforation.

Magnetization measurements were conducted on a Quantum Design MPMS3 SQUID magnetometer equipped with a 7 T magnet, operating in the temperature range 2–1000 K. Electrical resistivity, magnetoresistance, and specific heat were measured on a Quantum Design Physical Property Measurement System (PPMS 9T) equipped with a 9 T magnet and operating in the temperature range 2–400 K.

## Supporting Information Available

Explanation why the [111] direction, which is quite unusual direction for a tetragonal structure, was chosen in the HAADF-STEM experiments (PDF)
