# Supplementary material for: Crystal Structure and Ferromagnetism of the CeFe9Si4 Intermetallic Compound
Source: Inorg Chem. 2023 Apr 6;62(15):6169–80. doi: 10.1021/acs.inorgchem.3c00547 (PMC10114074; doi:10.1021/acs.inorgchem.3c00547)
Supplement: Supplementary file 1 — ic3c00547_si_001.pdf [file ic3c00547_si_001.pdf]

Supporting Information

Crystal structure and ferromagnetism of the $CeFe_9Si_4$ intermetallic compound

Primož Koželj,[1,2] Stanislav Vrtnik,[1] Justine Boutbien,[3] Jože Luzar,[1] Andreja Jelen,[1] Sorour Semsari Parapari,[1] Pascal Boulet,[3,§] Marie-Cécile de Weerd,[3] Gwladys Lengaigne,[3] Magdalena Wencka,[1,4] Vincent Fournée,[3] Julian Ledieu,[3] Sašo Šturm,[1] Janez Dolinšek[1,2,]*

[1] *J. Stefan Institute, Jamova 39, SI-1000 Ljubljana, Slovenia*

[2] *University of Ljubljana, Faculty of Mathematics and Physics, Jadranska 19, SI-1000 Ljubljana, Slovenia*

[3] *Institut Jean Lamour, UMR 7198 CNRS – Université de Lorraine, Campus Artem, 2 allée André Guinier, BP 50840, F-54011 Nancy, France*

[4] *Institute of Molecular Physics, Polish Academy of Sciences, Smoluchowskiego 17, PL-60-179 Poznań, Poland*

[§] Corresponding author. *E-mail address*: p.boulet@univ-lorraine.fr (P. Boulet).

* Corresponding author. *E-mail address*: janez.dolinsek@ijs.si (J. Dolinšek).

The reason why the [111] direction, which is quite unusual direction for a tetragonal structure, was chosen in the HAADF-STEM experiments, is the following. Since the investigated $CeFe_9Si_4$ sample was polycrystalline, there was a need to find low-indexed crystal orientations closest to the microscope's optical axis on the thinned crystals. This approach is somewhat different from studying single crystals, where a TEM lamella can be prepared according to the desired orientation for the analysis. The aim was to search for the lowest-index zone axis (ZA), i.e. [100], [110] and [111], in the microcrystals within the polycrystalline sample. [111] was the closest ZA for an appropriate structural analysis, where the atomic columns of interest could be clearly resolved in the HAADF-STEM images.

The $CeFe_9Si_4$ structure looks very similar along different ZA. The ZA in this experiment was definitely [111]. Although the [100] ZA is similar to the [111], the experimental diffraction pattern and the atomically resolved structure match well with the [111] direction (Fig. S1). Due to a different aspect ratio of the principal orthogonal ZA, only the [111] direction provides a perfect fit, as seen from the analyzed SAED patterns and the corresponding HAADF-STEM images.

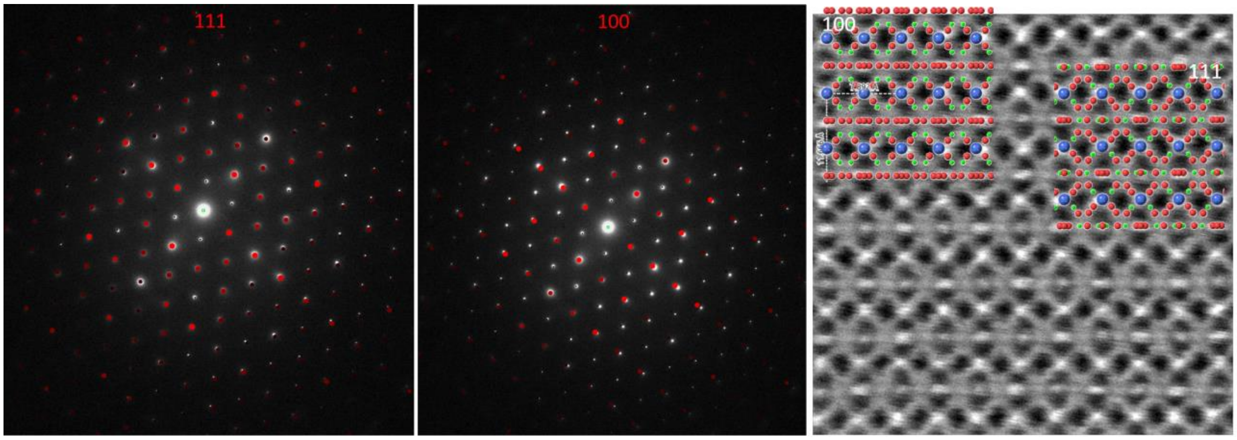

Fig. S1. Experimental SAED pattern with superimposed calculated diffraction pattern viewed along [111] (left panel) and [100] (middle panel) ZA for the $CeFe_9Si_4$ structure. Right panel:

Experimental HAADF-STEM lattice image and the overlaid atomic structures projected along [100] and [111] ZA.

The [111] and [100] directions of the $CeFe_9Si_4$ tetragonal structure correspond to the ⟨110⟩ directions of the cubic aristotype structure $NaZn_{13}$. This is the reason for the similarity of both directions of the tetragonal structure in Fig. S1 (due to the Fe and Si occupation, they are not identical as in the $NaZn_{13}$ structure, but similar), which is usually not typical for the tetragonal structures.
